# Prediction of promoters and enhancers using multiple DNA methylation-associated features

**DOI:** 10.1186/1471-2164-16-S7-S11

**Published:** 2015-06-11

**Authors:** Woochang Hwang, Verity F Oliver, Shannath L Merbs, Heng Zhu, Jiang Qian

**Affiliations:** 1 Department of Ophthalmology, Johns Hopkins School of Medicine, Baltimore, MD, 21205 USA; 2 Department of Pharmacology and Molecular Sciences, Johns Hopkins School of Medicine, Baltimore, MD, 21205 USA; 3 Center for High-Throughput Biology, Johns Hopkins School of Medicine, Baltimore, MD, 21205 USA; 4 The Sidney Kimmel Comprehensive Cancer Center,, Johns Hopkins School of Medicine, Baltimore, MD, 21025 USA; 5 Data Science for Knowledge Creation Research Center, Seoul National University, Seoul, 151-742 Korea

**Keywords:** DNA Methylation, Promoter, Enhancer, Regulatory region prediction, Feature selection, Support vector machine

## Abstract

**Background:**

Regulatory regions (e.g. promoters and enhancers) play an essential role in human development and disease. Many computational approaches have been developed to predict the regulatory regions using various genomic features such as sequence motifs and evolutionary conservation. However, these DNA sequence-based approaches do not reflect the tissue-specific nature of the regulatory regions. In this work, we propose to predict regulatory regions using multiple features derived from DNA methylation profile.

**Results:**

We discovered several interesting features of the methylated CpG (mCpG) sites within regulatory regions. First, a hypomethylation status of CpGs within regulatory regions, compared to the genomic background methylation level, extended out >1000 bp from the center of the regulatory regions, demonstrating a high degree of correlation between the methylation statuses of neighboring mCpG sites. Second, when a regulatory region was inactive, as determined by histone mark differences between cell lines, methylation level of the mCpG site increased from a hypomethylated state to a hypermethylated state, the level of which was even higher than the genomic background. Third, a distinct set of sequence motifs was overrepresented surrounding mCpG sites within regulatory regions. Using 5 types of features derived from DNA methylation profiles, we were able to predict promoters and enhancers using machine-learning approach (support vector machine). The performances for prediction of promoters and enhancers are quite well, showing an area under the ROC curve (AUC) of 0.992 and 0.817, respectively, which is better than that simply based on methylation level, especially for prediction of enhancers.

**Conclusions:**

Our study suggests that DNA methylation features of mCpG sites can be used to predict regulatory regions.

## Introduction

Transcriptional regulation plays an important role in most of biological processes. The interactions between transcription factors and regulatory regions, such as promoters and enhancers, are essential in transcriptional regulation. Therefore, identification of regulatory regions will provide mechanistic insight into various biological processes. Experimental and computational approaches have been developed to identify the regulatory regions on a genome-wide scale. For example, evolutionary conservation, sequence motifs, and clustering of transcription factor binding motifs (or cis-regulatory modules) can be used to predict regulatory regions [1-4]. However, these approaches are based purely on DNA sequences, which do not reflect the tissue-specific nature of the regulatory regions.

Recently, histone marks were measured on a genome-wide scale using the ChIP-seq technique [5-7]. These histone marks are good predictors for regulatory regions. For example, H3K4me1 is associated with active enhancers, while H3K4me3 is related to active promoters [8]. In addition, DNase I hypersensitivity sites (DHS) also localize to open chromatin regions, which are likely regulatory regions [9,10]. The ENCODE project has generated histone marks and DHS profiles in multiple cell lines and tissues [8,9,11,12], which provide valuable information to understand the organization and dynamics of the regulatory regions in the cells.

In this work, we propose to predict regulatory regions by utilizing the DNA methylation patterns. DNA methylation, the addition of a methyl group to the fifth carbon of a cytosine residue adjacent to a guanine (a CpG site), is a well-studied epigenetic modification. While DNA methylation is considered stable and heritable in differentiated somatic cells, it can also change dynamically during the lifespan of a cell and is susceptible to diet and other environmental influences [13,14]. DNA methylation is known to play a role in gene regulation. It is well accepted that DNA methylation in promoter regions represses the expression of the genes [15]. High-throughput technologies, such as whole genome bisulfite sequencing and array-based methods, have enabled the mapping of DNA methylation patterns on a genomic scale, identifying hundreds of millions of methylated cytosines [16-19].

DNA methylation-based approach has been developed to predict regulatory regions in recent work [20,21]. In these studies, low methylation regions are associated with distal regulatory elements, as they are enriched for active histone marks (e.g. H3K4me1), DNase-hypersensitive sites, and transcription factor binding sites (TFBS). In this study, we performed a comprehensive survey of mCpGs in regulatory regions and explore whether we can extract a set of DNA methylation dependent features besides the methylation level to improve the prediction. By examining these properties of the mCpG sites across different cell lines, we discovered that these sites did demonstrate specific genomic properties. Using these genomic features, we were able to predict regulatory regions using support vector machine approach. The paper is organized as follows. We first defined the positive and negative sets for regulatory region prediction. We then described the novel features derived from DNA methylation profiles. Finally, we utilized machine-learning approach (support vector machine) to predict regulatory regions (promoters and enhancers separately) based on the features we obtained. Our results demonstrate that the performance of the prediction based on multiple DNA methylation-associated features is better than the prediction solely based on methylation level.

## Results

### Selection and assessment of positive and negative datasets

We used the previously established definition of a regulatory region as determined by genome-wide histone modification signatures [8]. For example, H3K4me3 is known to be associated with promoters, while H3K4me1 with enhancers. Ernst *et al*. predicted different types of regulatory regions using a hidden Markov model. In this paper, we focus on two major regulatory regions: promoters and enhancers.

We also selected the same numbers of the mCpG sites in random genomic regions as negative datasets. To exclude the effect of differential methylation due to genomic location, we chose random genomic regions with the same relative distance to the nearest transcription start site (TSS) or exon-intron boundary for each type of regulatory regions (see Methods for details).

**Figure 1:**
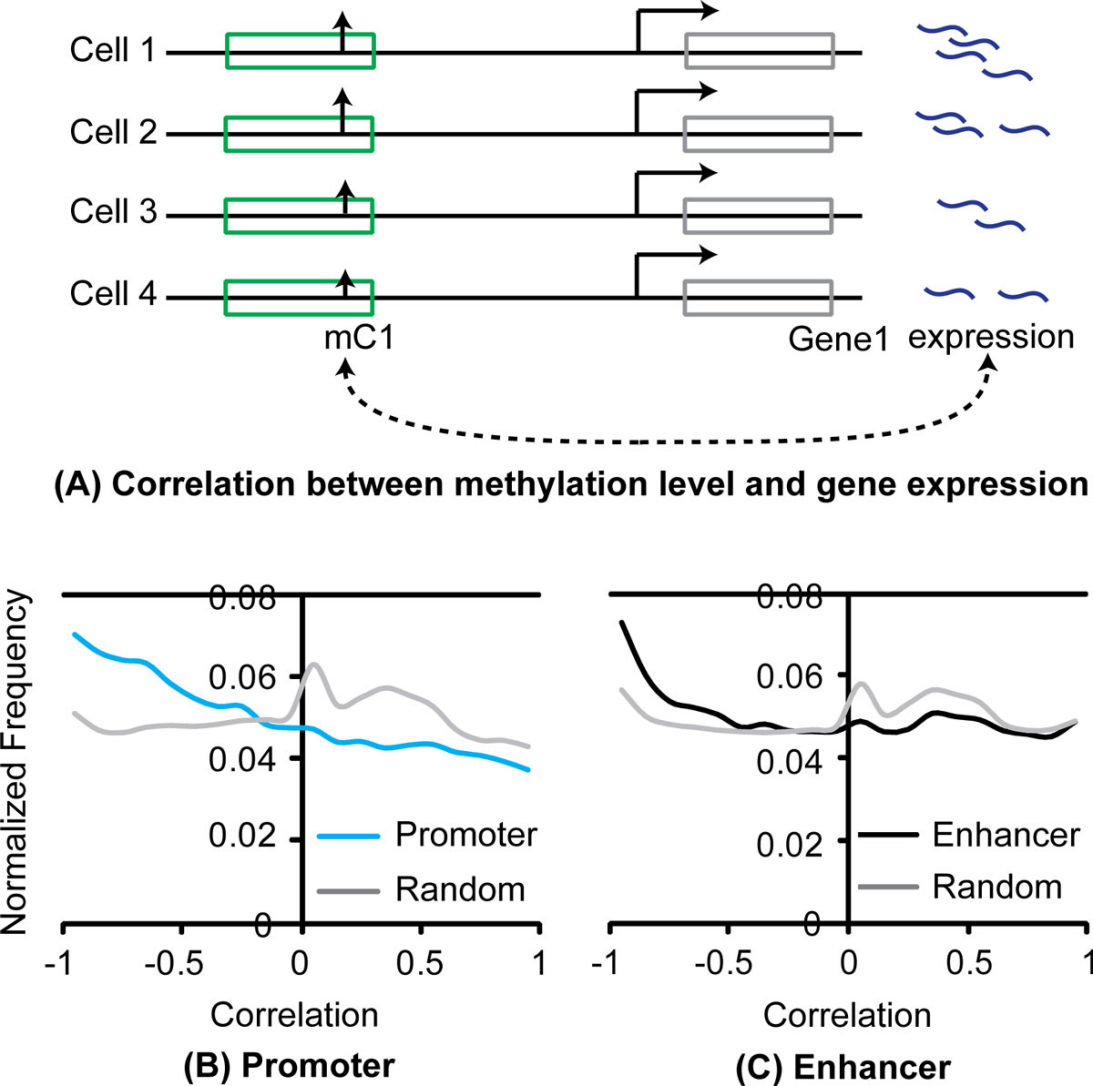
**The regulatory regions predicted by histone marks are likely to be functional.** (A) Correlation between methylation level of a mCpG and its target gene's expression. (B-C) Distribution of correlation between methylation level of mCpGs and their target genes' expression in regulatory regions and random regions. mCpGs in regulatory regions are hypomethylated.

To determine the quality of the regulatory regions determined by histone marks, we calculated the correlation between the methylation level of a mCpG site and the expression of its associated target genes (Figure 1A). The adjacent gene of a promoter was selected as its target gene. The predicted target genes for enhancers were obtained from Thurman *et al*. [9], which is based on the correlation of DNase I hypersensitivity activity between distal elements and promoters across cell lines. For each methylation site, Pearson correlation coefficient was used to compute the correlation between methylation level at a given site and the expression of its target gene across cell lines [22]. As comparison, the same correlation was calculated for the negative datasets.

Compared to the mCpG sites in random genomic regions, the methylation level of the mCpG sites in the regulatory regions was significantly more negatively correlated with the expression of the target genes (p < 1.0E-15; Kolmogorov-Smirnov test) (Figure 1B and Figure 1C). This is consistent with the notion that DNA methylation represses gene expression. We would like to point out that the conclusion still holds if we used Spearman's rank correlation, rather than Pearson correlation coefficient (Additional file 1: Figure. S1A, B).

Overall, our result suggests that the regulatory regions predicted by histone marks and the random genomic regions with similar relative genomic locations are of reasonable quality and are suitable to serve as the positive and negative sets of this study.

### mCpGs in regulatory regions are hypomethylated

**Figure 2:**
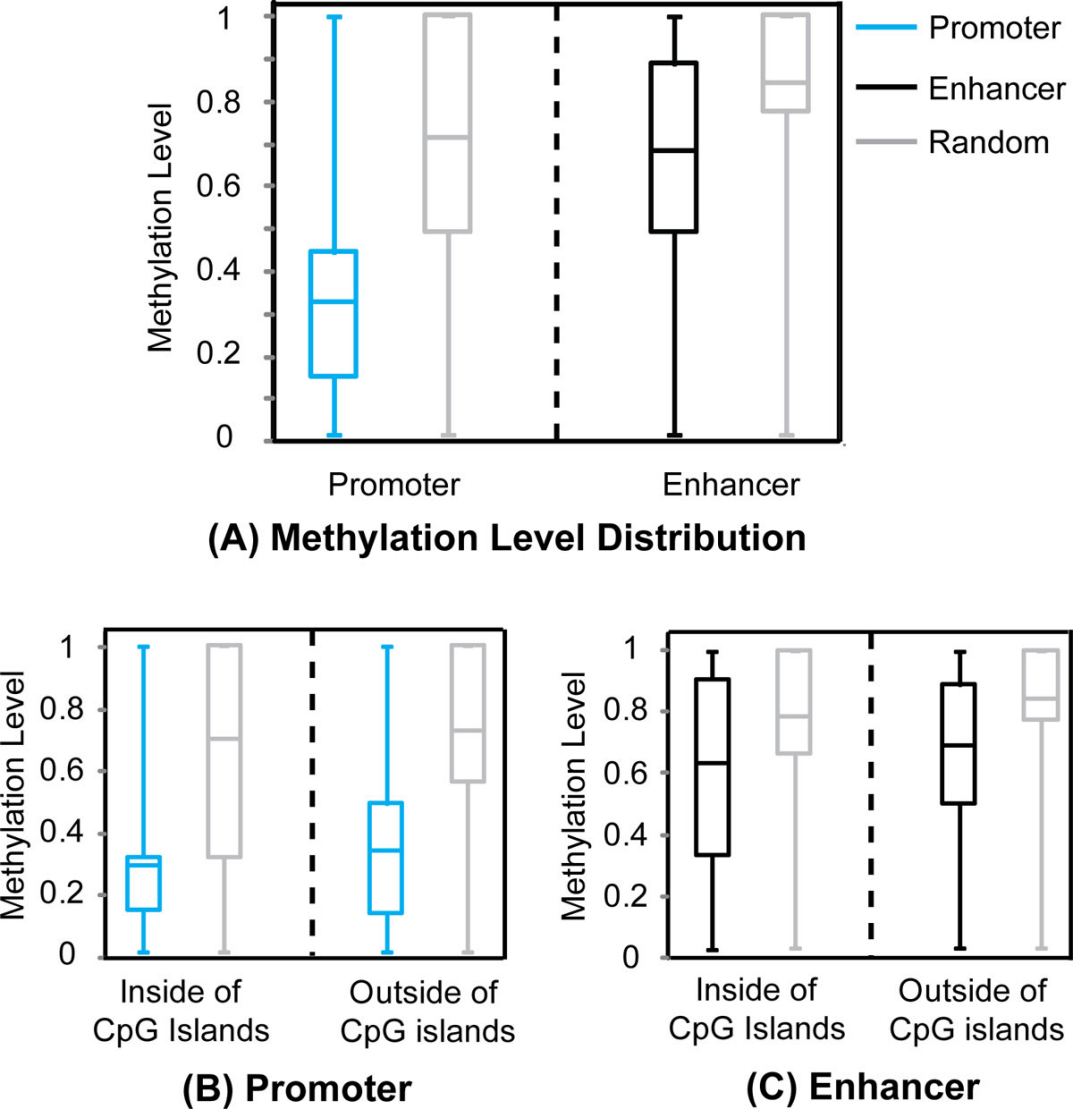
**CpG sites are hypomethylated in regulatory regions.** (A) Methylation level distribution of mCpGs in regulatory regions (blue and black) and random regions (grey). (B-C) Methylation level distribution of mCpGs inside and outside of CpG islands for regulatory regions (blue and black) and random regions (grey).

We then extract a series of DNA methylation-dependent features by comparing the methylation sites in the regulatory regions and random mCpG sites in the negative sets. We found that the methylation level of regulatory mCpG sites was significantly lower than the other mCpG sites (Figure 2). The average methylation levels of mCpGs in active promoters and the corresponding negative set are 0.34 and 0.72, respectively, showing statistically significant difference (p < 1.0E-15 based on Kolmogorov-Smirnov test). Similarly, the methylation levels of mCpGs in strong enhancers and the corresponding negative sets are 0.69 and 0.84, respectively (p < 1.0E-15 based on Kolmogorov-Smirnov test). These results persisted when CpG islands in the regulatory regions and the corresponding random regions were excluded, indicating that the low methylation level in regulatory regions was not due to the overlap with CpG islands, which are generally hypomethylated (Figure 2B, 2C). Interestingly, regulatory regions overlapping with CpG islands demonstrated lower methylation levels than regulatory regions outside of the CpG islands (p < 1.0E-15 based on Kolmogorov-Smirnov test; Figure 2B, 2C), suggesting that those CpG islands playing a regulatory role have lower methylation levels. Overall, our observations suggested that hypomethylation is a key feature of regulatory regions, independent of the relative location to a CpG island.

### Hypomethylation in regulatory regions extends across a long range

**Figure 3:**
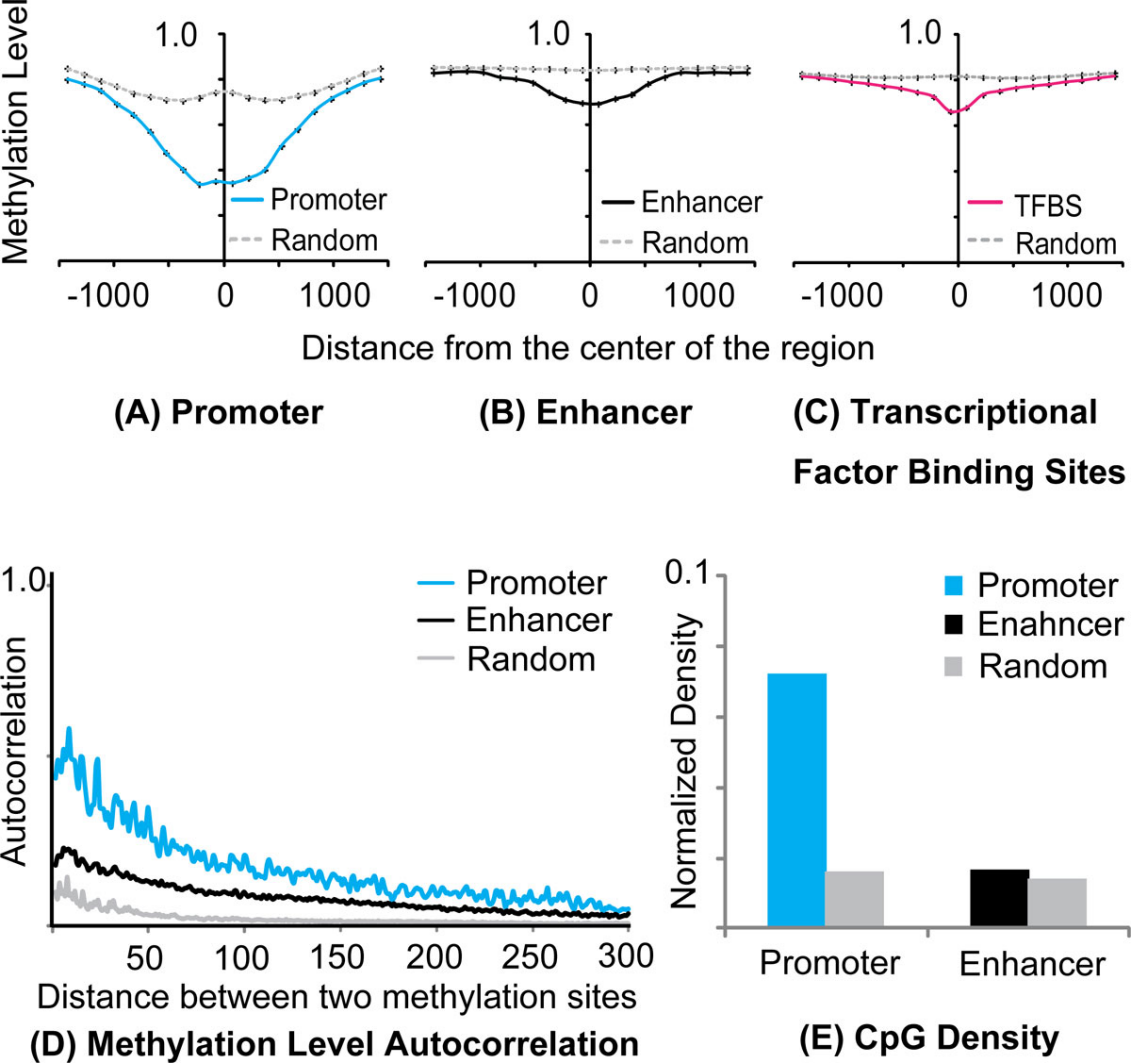
**The hypomethylation in regulatory regions extends a long range.** (A-C) Average methylation level of mCpGs in regulatory regions and random regions. The regions were aligned on their center position. × axis is the distance from the center of the regions. Y axis is the average methylation level. (D) Autocorrelation between two mCpGs in regulatory regions and random regions. × axis is distance between two mCpGs. (E) CpG density of regulatory regions and random regions.

Within a methylated regulatory region, the lowest methylation level is found centrally within the region, and this hypomethylation extends across a long range (Figure 3). For example, the average methylation level of mCpGs at the center of active promoters (0.34) gradually increases across the promoter region and reaches a plateau (0.82) at ~1500 bp away, suggesting that hypomethylation is not localized to a narrow genomic region (Figure 3A). The extended hypomethylated regions were also apparent within strong enhancers (Figure 3B). Strikingly, the extended hypomethylation was also observed within transcription factor (TF) binding sites, which are generally more localized to 10-20 bp regions. Using ChIP-seq and predicted data for several TFs [23], we found that the methylation levels were lowest in the center of TF binding sites and reached background level by 1500 bp (Figure 3C), despite the fact that the majority of binding sites are less than 20 bp in length.

### Methylation levels of neighboring regulatory mCpG sites are highly correlated

One explanation of the extended hypomethylation is correlation between neighboring regulatory mCpG sites. To test this hypothesis, we calculated the autocorrelation of methylation profiles within regulatory regions. Specifically, we computed the correlation of methylation levels at two methylation sites as a function of their genomic distances (Figure 3D, see Methods for detail). As expected, mCpG sites within close proximity showed higher correlation than those located distally. Based on the autocorrelation of methylation profile, we observed that the correlation of methylation between mCpG sites was significantly stronger in regulatory regions than in non-regulatory regions (p < 1.0E-15 based on Kolmogorov-Smirnov test; Figure 3D). In regulatory regions, the correlation of methylation levels extended across distances of up to 326 and 231bp for promoters and enhancers, respectively, whilst in random genomic regions the correlation disappeared by 41bp (see also Additional file 1: Figure S1C).

We speculated that a high density of CpG sites could be the underlying mechanism for correlation between methylation levels, as sites in close proximity might be co-regulated by DNA methyltransferases. We examined the CpG densities in the regulatory and random regions and found that regulatory CpG sites were much denser than those in random regions, especially within active promoters (Figure 3E). This observation was true even if the CpG islands in these regions were excluded (Additional file 1: Figs S1D, E). Thus, the hypomethylation in regulatory regions and/or TF binding sites can be attributed to both the high correlation between methylation levels and the high CpG density within these regions.

### Inactive regulatory regions are hypermethylated

**Figure 4:**
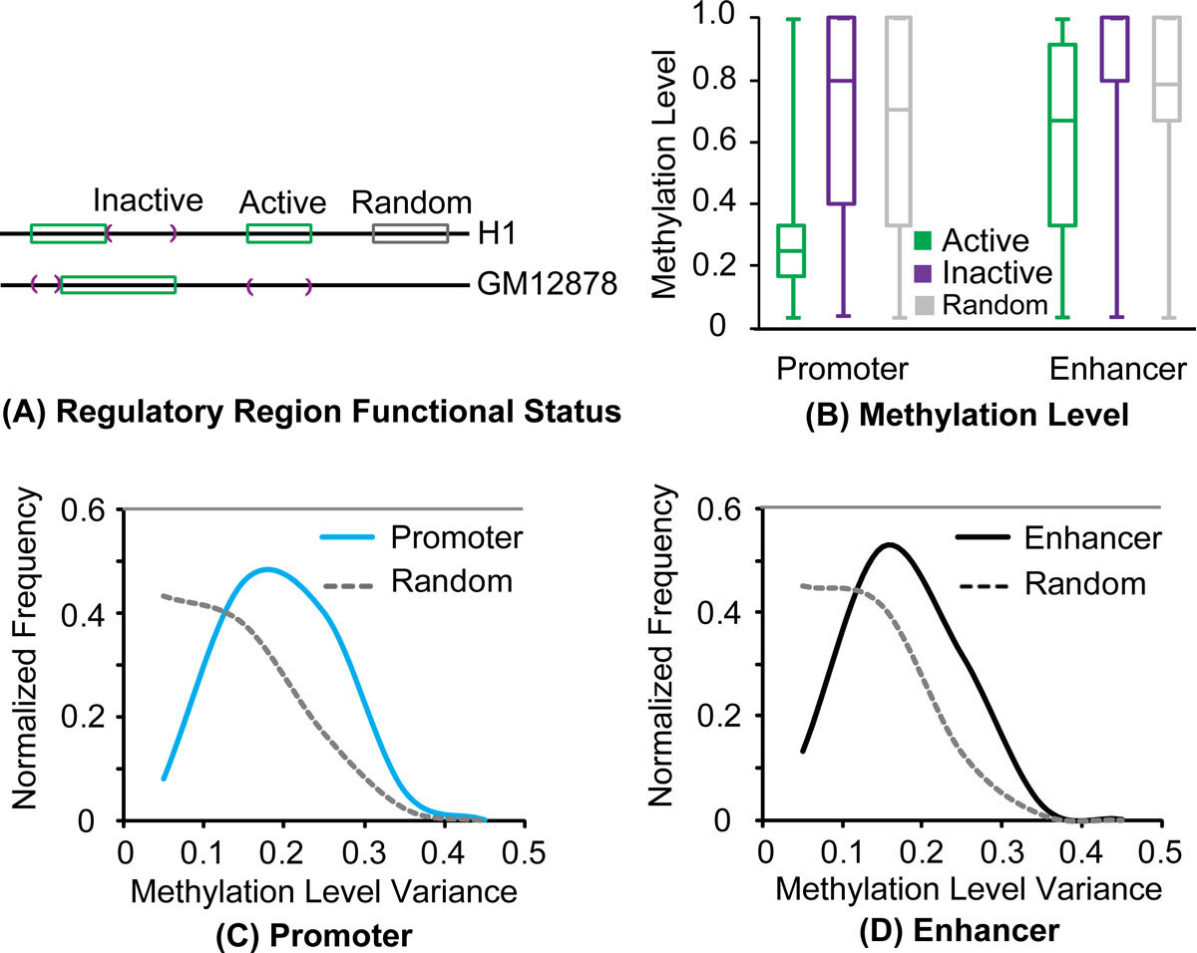
**Methylation level changes with the regulatory region status.** (A) Functional status changes of a genomic region among different cells. A region can be active (green) in a cell (H1) while inactive (purple) in another cell (Gm12878). (B) Methylation level distribution of mCpGs inside of CpG islands in active, inactive and random regions. (C-D) Methylation level variance of mCpGs in regulatory and random regions among 15 cell lines.

Epigenetic marks in regulatory regions differ between different cell types. For example, in a given cell line a genomic region can be an active promoter enriched for active histone marks (*e.g*. H3K4me3, H3K9ac, and H3K27ac), while the same region may be inactive in another cell line due to the absence of such histone marks. To address the role of histone modifications within regulatory regions, we compared changes in the methylation level of the regulatory mCpG sites to changes in histone mark status. The methylation levels between two cell lines (H1 and GM12878) were examined, as genome-wide histone modification and methylation data were available for both cell lines. We defined three genomic regions for each cell line: transcriptionally active, transcriptionally inactive, and random (background) regions. For example, the genomic regions predicted as regulatory regions in H1 cell were defined as 'active' in H1 cell. The genomic regions that were predicted as regulatory regions in GM12878 cell, but not in H1 cell were categorized as 'inactive' in H1 cell. These "inactive" regions represent the genomic regions that are not enriched by transcriptionally active histone marks in H1, but have the potential to become transcriptional active in other cell lines. As comparison, we also selected a set of genomic regions that do not overlap with any regulatory region in both H1 and GM12878 (see details in Methods and Figure 4A). The mCpGs methylation levels inside active, inactive, and random regions in H1 cell were compared.

Using our comparative analysis between H1 and GM12878 cells, we found that the methylation levels at mCpG sites within inactive promoters and enhancers resembled neither the active nor the background (random region) levels (Figure 4B). Interestingly, the methylation levels in inactive regulatory regions were even higher than background levels obtained from random regions (p < 1.0E-15; Kolmogorov-Smirnov test) (Figure 4B). This phenomenon was robust as it was present when we compared H1 cells with other different cell lines (additional file: 2: Figure S2 A-E). Furthermore, the finding still held if we grouped the regulatory regions based on whether they overlap with CpG islands (Figure 4B, additional file 2: Figs S2 A-E, 3: Figure. S3). Finally, rather than using predicted active and inactive regulatory regions, if we used the raw histone marks (e.g. H3k9ac), we made the same observation in multiple cell lines (Additional file 4: Figure S4). Our findings demonstrate that inactive regulatory regions show hypermethylation relative to the genomic background, distinguishing inactive regulatory regions from background methylation.

### Methylation level in regulatory regions can vary considerably between cells

The difference in methylation levels between active and inactive regions suggested that regulatory mCpG sites might have a greater range of possible methylation levels than other mCpGs. To test this hypothesis, we calculated the variability of each mCpG site across the cell lines whose genome-wide methylation profiles are publically available [22]. The variance of the methylation levels for the regulatory regions predicted in H1 cells were then compared to those from random genomic regions (Figure 4C, 4D). About 40% of the regulatory mCpG sites have a variance larger than 0.2, whereas only 15% of mCpG sites in other genomic regions showed the same variance, suggesting that the regulatory mCpG sites have a larger range of potential methylation levels.

We then directly related the methylation level of mCpGs to the regulatory state (active versus inactive). For those mCpG sites that were present in an active regulatory region in both cell lines, the difference in methylation level between the two cell lines was small (Additional file 2: Fig. S2F). In fact, the vast majority (~75%) of the regulatory mCpG sites showed very small methylation level differences in the range of -0.1 to 0.1 between the two different cell lines. This difference was similar to the difference in methylation levels of mCpG sites in random genomic regions. In contrast, the regulatory mCpG sites present in both active and inactive regulatory regions in two cell lines showed a greater methylation difference; less than a half (42%) of the active-inactive regulatory regions had methylation level differences between -0.1 and 0.1. Taken together, the large range of potential methylation levels in the regulatory regions can be associated with the status changes of the regulatory regions.

### Distinct sequence motifs are associated with regulatory mCpG sites

**Figure 5:**
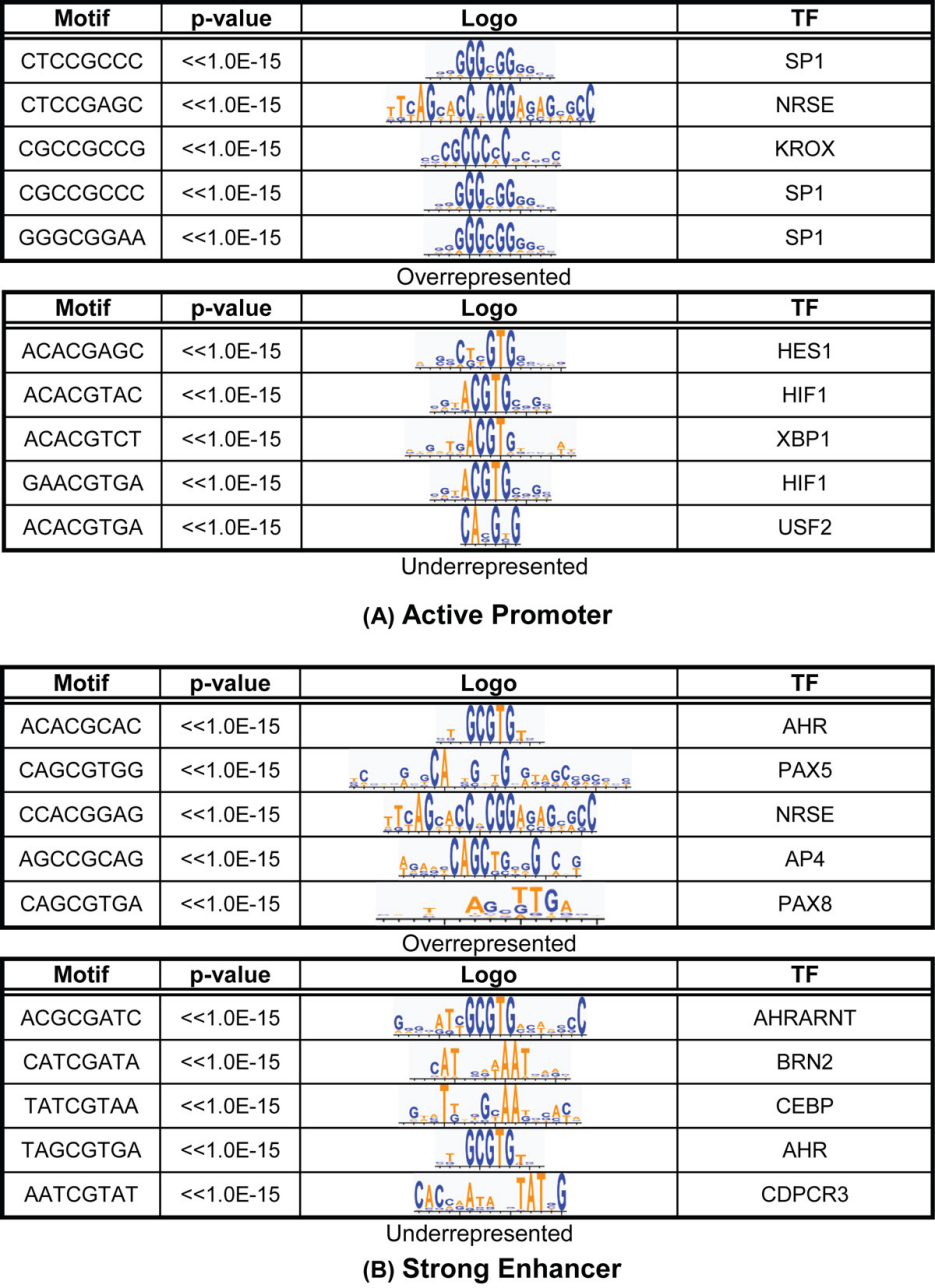
**DNA Motifs are associated with CpG sites in regulatory regions.** 5 most significantly overrepresented and underrepresented sequence motifs surrounding mCpGs in regulatory regions. Third and fourth column show the sequence logos and the names of the best matched transcriptional factor binding sites. (A) Active promoter (B) Strong enhancer

Since DNA motifs were used to predict enhancers, we also explore to identify the DNA motifs associated with mCpG sites in regulatory regions. The rationale is that DNA methylation is believed to play a crucial role in the transcription process by directly interfering with TF binding to the regulatory regions [24]. Therefore, we hypothesize that sequence motifs around the mCpGs in regulatory regions were more likely to be TF binding sites and would serve as another informative feature distinguishing regulatory mCpG sites. We examined all 8-mer sequences with a CpG in the center and compared the occurrence of each motif in the regulatory regions and in the random regions. The statistical significance of each motif was evaluated, and both overrepresented and underrepresented motifs were identified. In total, 86 and 194 8-mers were significantly overrepresented in the active promoters and the strong enhancers, respectively, compared to background (p < 1.0E-5). Additionally, we found 104 and 86 significantly underrepresented 8-mers in the active promoter and the strong enhancers (p < 1.0E-5). Figure 5 lists the top 5 most significantly overrepresented and underrepresented 8-mers in the regulatory regions (see additional file 5: Fig. S5 for additional motifs). These motifs were also compared with known transcription factor consensus sequences (5: Figure. S5).

Interestingly, the nucleotide composition of the overrepresented 8-mers was different from that of the underrepresented 8-mers. For example, the GC content of overrepresented 8-mers obtained from the promoters and enhancers are 0.68 and 0.60, respectively. In contrast, the GC contents of the underrepresented 8-mers from the promoters and enhancers are only 0.47 and 0.31, respectively. This distinct nucleotide composition difference became more significant when considering only the 2 bases directly adjacent to the CpG sites. Over 77% and 68% of the overrepresented 8-mers in the promoters and enhancers, respectively, had either cytosines or guanines as a direct neighbor of the CpG site. In contrast, only 31% and 28% of the underrepresented 8-mers in these two types of regulatory regions had either cytosines or guanines at these positions. Cytosines or guanines were simultaneously observed in both direct neighbors of the overrepresented motifs (56% and 45% in the promoters and enhancers, respectively), which occur much less frequently in the underrepresented motifs (13% and 11% in the promoters and enhancers, respectively).

### Regulatory regions are predictable

Distinct genomic features of the regulatory mCpGs, which distinguish them from other mCpGs in negative sets, were used to predict regulatory regions. We predicted the regulatory regions using these features, including methylation level, CpG density, autocorrelation of methylation levels, variance of methylation levels among different cell lines and sequence motifs (significance *(-log(P)*) of the occurrence of 8-mer sequence motifs surrounding the mCpGs). The computation of these features is described in detail in Methods section. As comparison, we predicted the regulatory regions solely based on methylation level.

**Figure 6:**
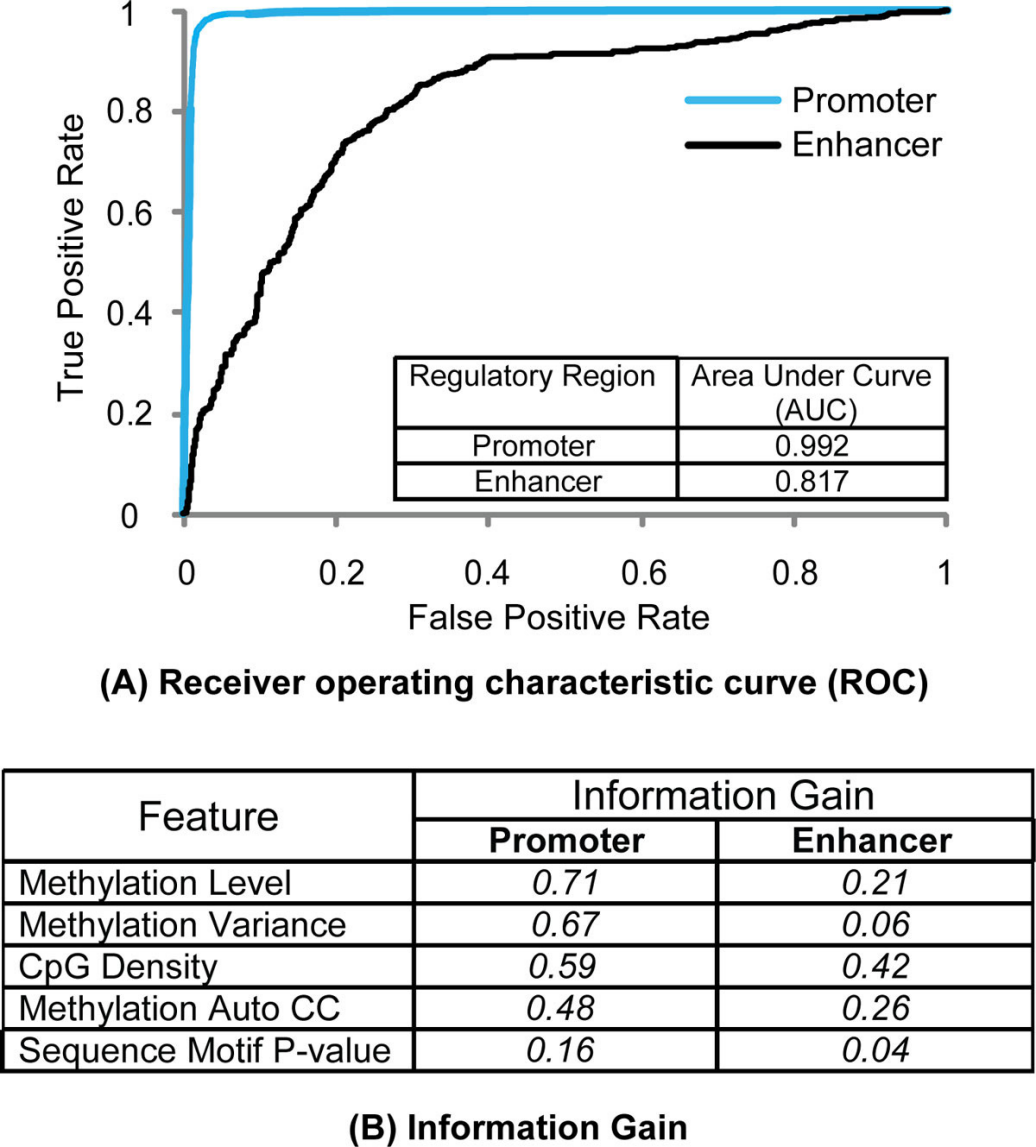
**Regulatory regions can be predicted using the features derived from DNA methylation profiles.** (A) Receiver operation characteristic curve (ROC) of regulatory region prediction using methylation profile in the regions. Support vector machine (SVM) was used. (B) Information gain of features in the prediction.

Support vector machine (SVM) was used to classify regulatory and non-regulatory regions. SVM finds a set of hyper planes that separates data points into a set of classes in high dimensional feature space. To distinguish between regulatory and random genomic regions, 10-fold cross validation was used to measure the classification performance, in which 90% of regions were used to train the model, and the remaining 10% of regions were used as test dataset (see details in Methods). Figure 6A illustrated the performance of the prediction for active promoters and strong enhancers using a Receiver Operating Characteristic (ROC) curve. The area under the ROC curve (AUC) was used to depict the performance of a binary classification. Active promoters and strong enhancers were predicted quite well, showing an AUC of 0.992 and 0.817, respectively (Figure 6A). Perhaps not surprisingly, if we only used the methylation levels as the feature for prediction, the performance is lower, with AUC of 0.985 and 0.692 for promoters and enhancers, respectively. The prediction for enhancers is significantly improved with the additional features.

Some features showed more significant contribution to the prediction than other features. Therefore, the ability of each feature to discriminate regulatory and non-regulatory regions was analyzed by the information gain of each feature (Figure 6B). Information gain (IG) of a given feature *F *with respect to the classes (e.g., regulatory or random regions) is the entropy reduction of the sample set when we know the feature *F *(see Methods in detail). Interestingly, the most informative features are different when predicting promoters and enhancers. For the promoters, the features showing the largest information gain are methylation level and methylation variance, while for enhancers, the most useful features are CpG density and methylation autocorrelation (Figure 6B). Some features show strikingly different contribution in predicting promoters and enhances. For example, the feature of methylation variance is quite informative in promoter prediction, with information gain of 0.67, while it has limited contribution when predicting enhancers, with information gain of 0.06. This result suggests that a set of methylation-associated features are needed for predicting regulatory regions and these features have different predictive power for promoters and enhancers.

## Discussion

Although low methylation has been found to be associated with regulatory regions [16,21], we found that a low methylation level was not sufficient to predict regulatory regions, especially for enhancers. Additional features of mCpG sites were required to predict regulatory regions, and we were able to elucidate some of these features and successfully use them for prediction. For example, in regulatory regions, we found that a low methylation level extended across a range that was often longer than 1000 bp, and that the correlation of methylation levels between methylation sites was much stronger than outside of regulatory regions. Furthermore, we found a larger variation in methylation levels within regulatory regions compared to non-regulatory regions. Therefore, our work provides novel insights regarding the DNA methylation status in regulatory regions.

Since CpG islands are often lowly (or not at all) methylated and are considered to play an important role in gene regulation, the hypomethylated state is regarded to be important in positive gene regulation. In this study, we found that regulatory mCpG sites demonstrate distinct features beside hypomethylation. Specifically, we found that the methylation levels are highly correlated between neighboring mCpG sites in regulatory regions. Furthermore, when regulatory regions are inactive in other cell types, these methylation levels did not simply return to the background level but were instead hypermethylated, suggesting that in regulatory regions, a higher level of methylation is required to maintain an inactive state.

Correlation of the DNA methylation status of neighboring CpG sites [25,26] has been previously observed; however, in these studies, the correlation was not analyzed in the context of regulatory regions. For example, Eckhardt *et al*. found an overall correlation between neighboring CpG sites in the human genome [25]. Our work revealed that the correlation primarily stems from the regulatory regions as the correlation in regulatory regions was much stronger than that in random genomic regions.

The overrepresented 8-mer motifs in regulatory regions are predicted as potential transcription factor binding sites. Our prediction suggested a distinct set of transcription factors might interact with these motifs in a methylation-dependent fashion since the overrepresented motifs had a higher GC content. While the chromatin structure was previously considered as one mediator of transcription factor-DNA interaction, our finding indicates that DNA methylation can also serve as a "switch" for protein-DNA interactions [27,28]. Indeed, the chromatin structure and DNA methylation can influence each other. For example, nucleosome occupancy can direct DNA methylation [29,30], and in some cases, DNA methylation can determine nucleosome occupancy [31].

In this study, we demonstrated that regulatory regions are predictable by their methylation patterns; however, our prediction did not perfectly separate regulatory and non-regulatory regions, especially at enhancers. One possible reason is that we did not have sufficient methylome datasets for the prediction model. Since these behaviors required methylation levels from multiple cell types, additional methylation data from a range of cell types should help to better describe these distinctive behaviors. We expect such datasets will become available in near future and will enable better prediction of the enhancers. Another possibility is that DNA methylation is also associated with other functional elements other than promoters and enhancers. For example, recent studies suggested that DNA methylation is also involved in alternative splicing regulation [32-34]. We need additional information to distinguish different types of functional elements to improve our prediction.

## Conclusion

We proposed a set of novel methylation associated features that are informative to predict regulatory regions. These features greatly improve the prediction compared to the prediction solely based on methylation level. Our results suggest that the regulatory "grammar" is encoded in complex DNA methylation patterns and identification of these features will provide biological insight on the methylation-mediated gene regulation.

## Methods

### Data

Human DNA methylation data as measured by bisulfite sequencing or Illumina 450k array available for 15 cell lines was obtained from SALK and ENCODE databases. The regulatory regions predicted from chromatin marks were downloaded from ENCODE database [35]. Gene expression data by RNA-Seq, available in 4 cell lines, was imported from SALK database [16]. RPKM (Reads per kilo base per million) values was used for transcripts' expression [36]. Transcription factor binding is based on ChiP-seq or computational predictions [23,35]. Gene annotation from UCSC Genome database was used [37].

### CpG sites in the study

The methylation level of a CpG site was measured as the ratio of the number of methylated cytosines to the total number of sequences on that position in the bisulfite sequencing data. To take into account only the sites with reliable measurement, we only consider the CpG sites covered with more than 4 sequences.

### Random region selection

**Figure 7:**
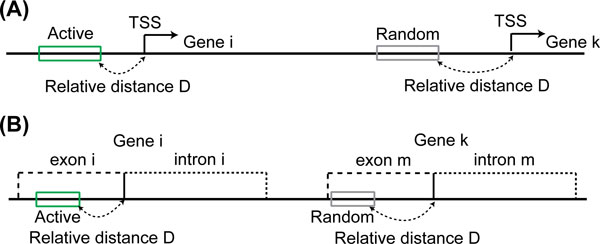
Schematic view of random region selection

Only the mCpGs in the coding regions and the upstream regions of genes were considered, where the upstream region of a gene was defined as the 10 kb region upstream of the gene transcription start site (TSS). The immediate downstream gene was classified as the target gene for those identified mCpGs. To exclude the effect of differential methylation due to different genomic regions, we selected random regions with similar genomic positions as mCpG sites. For a regulatory region in the upstream region, we selected the corresponding random regions with the same relative distance to the TSS (Figure 7A). Here the relative distance to the TSS was measured as the actual distance normalized by the total length of the coding region and the upstream region of the gene. For a regulatory region in an exon or an intron region, we selected the corresponding random regions with the same relative distance to the nearest boundary of the exon or intron of the target gene. Here the relative distance was normalized by the length of the exon or intron, depending on whether the region of interest is on exon or intron (Figure 7B). 1000 random region selections for each regulatory region were performed in this study.

### Autocorrelation of methylation profile

Methylation level autocorrelation was computed as follows:

(1) $$r_{k}=\frac{\sum_{i=1}^{N-k}\left(x_{i}-\bar{x}\right)\left(x_{i+k}-\bar{x}\right)}{\sum_{i=1}^{N}{\left(x_{i}-\bar{x}\right)}^{2}}$$

where *x_i _*is methylation level of a mCpG at position *i, x_i+k _*is methylation level of the mCpG distant *k *nucleotides from position *i*, and $\bar{x}$ is mean methylation level of the mCpGs in all regions of interest. We considered the autocorrelation disappeared when the value reached 0.05.

### CpG density and CG content

CpG density was calculated as the number of CpGs in a region normalized by its length. CG content in a region was measured as the number of cytosines and guanines in the region normalized by its total length.

### Sequence motif discovery

Only the 8-mer sequences with CpG in the center were considered. An 8-mer and its reverse complement were counted as the same motif. In theory, we have total 2080 possible 8-mers with CpG in the center. For each motif, we calculated the occurrences of the motif in regulatory regions (either promoter or enhancer), and compared the occurrences of the same motifs in the random genomic regions. P-value for each 8-mers was calculated based on binomial distribution using the occurrence probability in the random regions as background probability.

(2) $$pvalue=1-\sum_{i=0}^{k}\left(\begin{array}{c} n\\ i \end{array}\right)p^{i}{\left(1-p\right)}^{n-i}$$

where *p *is probability that an 8-mer is found in the random regions, and *k *is the number of occurrences of the 8-mer of interest and *n *is the number of all 8-mers in the regulatory regions. P-value was corrected for multiple testing using Bonferroni method.

### Regulatory region prediction

Support Vector Machine (SVM) was used to predict regulatory regions based on the genomic features of the mCpGs in the regions. To apply SVM to our dataset, a number of features that represent the entities (regions) in the dataset should be identified and transformed into feature vectors, i.e. multi-dimensional vectors in which each element is a selected feature. SVM builds a set of hyperplanes that separate the entities into specified classes utilizing the provided feature vectors. In this research, the test data set for prediction includes the predicted regulatory regions and the same number of random regions generated as we described in the previous section. Five features were used to form the feature vector, including mean methylation level, mean methylation variance among 15 cell lines, mean methylation level autocorrelation between two mCpGs, CpG density, and 8-mer sequence motif P-value around mCpGs in a genomic region. 10-fold cross validation was used to measure the prediction accuracy. In k-fold cross validation, the dataset is randomly partitioned into k equal size of subsets. k-1 subsets are used to train the prediction model and the remaining 1 subset is used to test the model. This cross validation process is repeated k times for each subset. For the SVM, polynomial kernel with the soft margin of 10 and the degree of 2 was used. The area under the ROC curve (AUC) was used to evaluate the prediction performance.

### Information gain

Contribution of a feature *F *in the classification for a sample set *S *was calculated as the information gain of *S *given the feature *F*, i.e., the difference between the entropy of *S *without any feature knowledge and the entropy of *S *given the feature *F*.

(3) $$IG\left(S,F\right)=H\left(S\right)-H\left(S|F\right)=H\left(S\right)-\underset{v\in values\left(F\right)}{\sum }\frac{\left|S_{v}\right|}{\left|S\right|}H\left(S_{v}\right)$$

*S *is a set of samples, *F *is a feature, *IG(S,F) *is information gain for a set *S *given a feature *F, values(F) *is the set of all possible values of an attribute *F, S_v _*is the subset of *S *that has value *v *for an attribute *F*, and *H(S) *is entropy of a set *S *defined as follows.

(4) $$H\left(S\right)=-\underset{i}{\sum }p_{i}{\text{log}}_{2}\left(p_{i}\right)$$

where *p_i _*is the probability that a sample in *S *is classified as class *i*. Discretization was adapted for continuous attributes.

## Competing interests

The authors declare that they have no competing interests.

## Authors' contributions

WH performed bioinformatics analysis and drafted the manuscript. VO provided experimental data and drafted the manuscript. SM participated in its design, and drafted the manuscript. HZ participated in its design, and drafted the manuscript. JQ conceived the study, participated in its design, and drafted the manuscript. All authors read and approved the final manuscript.

## Supplementary Material

Additional file 1 **Figure S1**. Click here for file

Additional file 2 **Figure S2**. Click here for file

Additional file 3 **Figure S3**. Click here for file

Additional file 4 **Figure S4**. Click here for file

Additional file 5 **Figure S5**. Click here for file

## Declarations

The publication costs for this article were funded by the corresponding author.

This article has been published as part of *BMC Genomics *Volume 16 Supplement 7, 2015: Selected articles from The International Conference on Intelligent Biology and Medicine (ICIBM) 2014: Genomics. The full contents of the supplement are available online at http://www.biomedcentral.com/bmcgenomics/supplements/16/S7.
