# Supplementary material for: Prediction of promoters and enhancers using multiple DNA methylation-associated features
Source: BMC Genomics. 2015 Jun 11;16(Suppl 7):S11. doi: 10.1186/1471-2164-16-S7-S11 (PMC4474542; doi:10.1186/1471-2164-16-S7-S11)
Supplement: Additional file 1 — Figure S1. [file 1471-2164-16-S7-S11-S1.pdf]

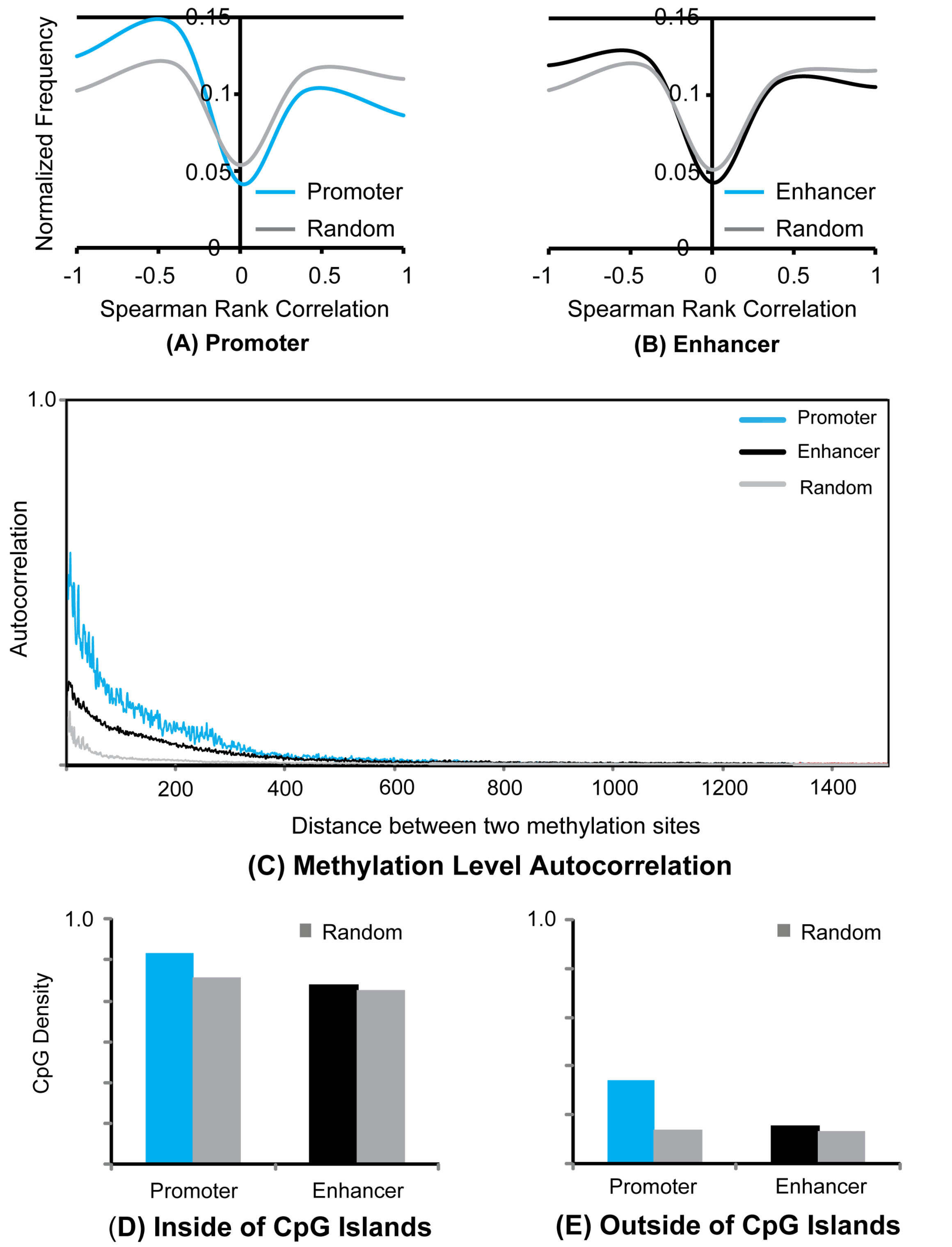

Figure S1 (A-B) Spearman's rank correlation between methylation level of a mCpG and its target gene's exression for promoters and enhancers. (C) Autocorrelation of methylation levels between two mCpGs in regulatory regions and random regions for further distance (1500bp). (D-E) CpG density of the regions inside and outside of CpG islands.
