# Supplementary material for: Prediction of promoters and enhancers using multiple DNA methylation-associated features
Source: BMC Genomics. 2015 Jun 11;16(Suppl 7):S11. doi: 10.1186/1471-2164-16-S7-S11 (PMC4474542; doi:10.1186/1471-2164-16-S7-S11)
Supplement: Additional file 2 — Figure S2. [file 1471-2164-16-S7-S11-S2.pdf]

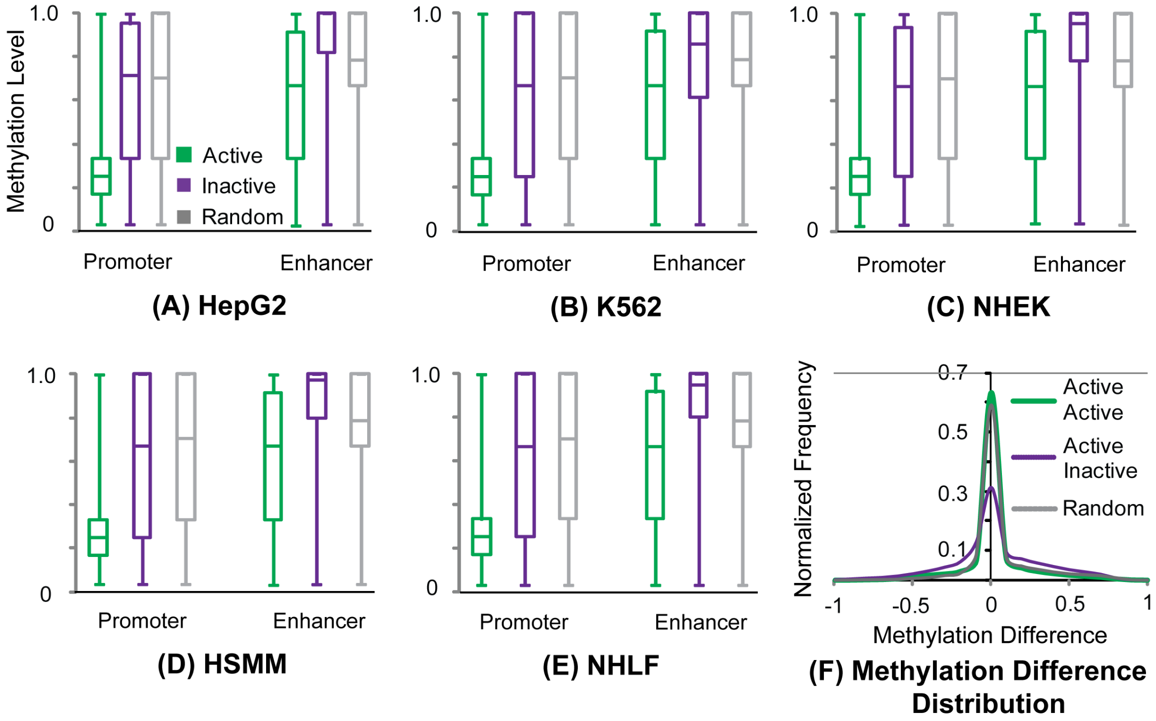

Figure S2 (A-E) Methylation level distribution of mCpGs inside of CpG islands in active, inactive, and random regions. (F) Methylation difference of mCpGs in the regions with different functional status. For example, an Active-Inactive region represents the region predicted as active in GM12878 cell but inactive in H1 cell.
