# Supplementary material for: Prediction of promoters and enhancers using multiple DNA methylation-associated features
Source: BMC Genomics. 2015 Jun 11;16(Suppl 7):S11. doi: 10.1186/1471-2164-16-S7-S11 (PMC4474542; doi:10.1186/1471-2164-16-S7-S11)
Supplement: Additional file 3 — Figure S3. [file 1471-2164-16-S7-S11-S3.pdf]

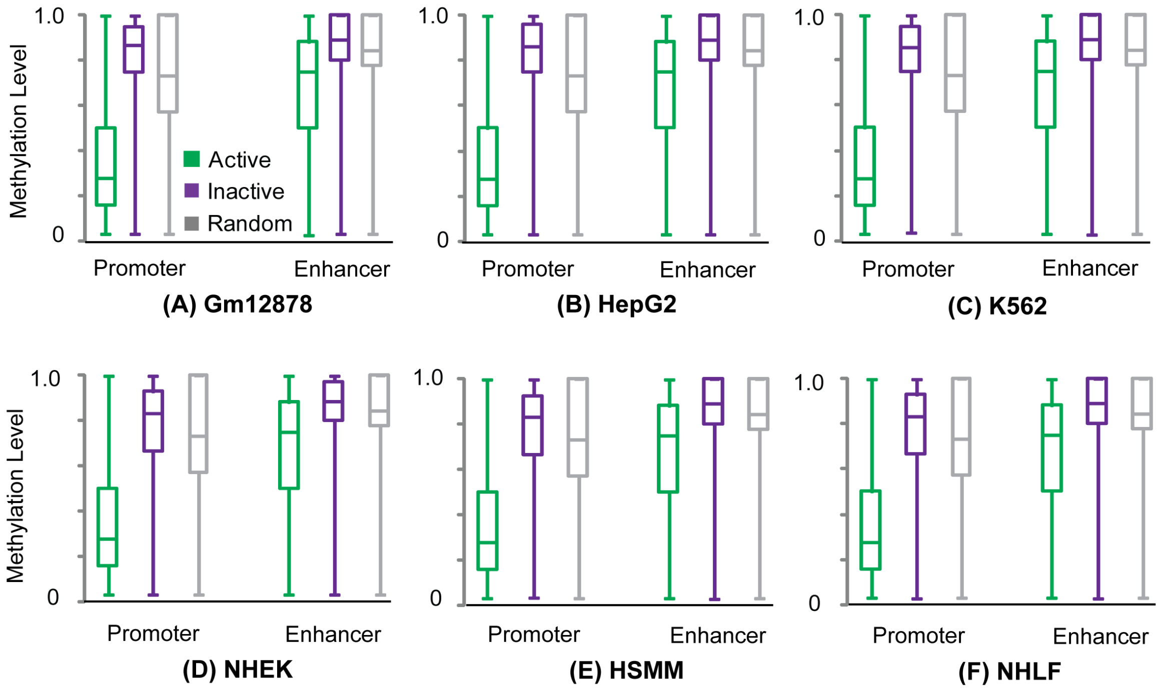

Figure S3 Methylation level distribution of mCpGs outside of CpG islands in active, inactive, and random regions. "Active" regions are the regions that are active in H1 cell. "Inactive" regions are the regions that are active in another cell but inactive in H1 cell.
