# Supplementary material for: Prediction of promoters and enhancers using multiple DNA methylation-associated features
Source: BMC Genomics. 2015 Jun 11;16(Suppl 7):S11. doi: 10.1186/1471-2164-16-S7-S11 (PMC4474542; doi:10.1186/1471-2164-16-S7-S11)
Supplement: Additional file 4 — Figure S4. [file 1471-2164-16-S7-S11-S4.pdf]

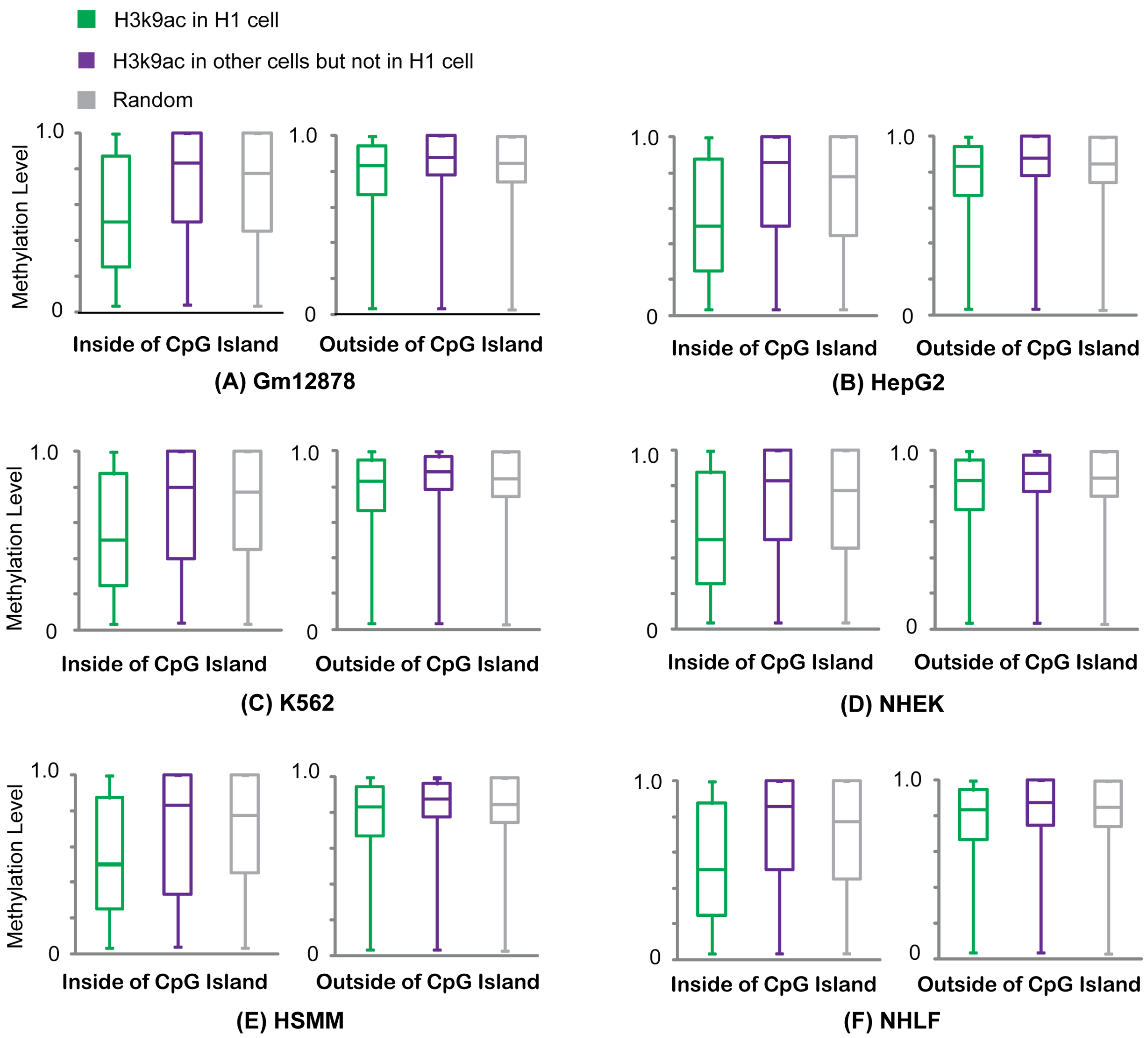

Figure S4 Methylation level distribution of mCpGs inside and outside of CpG islands in active, inactive, and random regions. “Active” regions are the histone marks (H3k9ac) peak regions presented in H1 cell. “Inactive” regions are the histone marks peak regions presented in another cell but not in H1 cell.
