# Supplementary material for: Prediction of promoters and enhancers using multiple DNA methylation-associated features
Source: BMC Genomics. 2015 Jun 11;16(Suppl 7):S11. doi: 10.1186/1471-2164-16-S7-S11 (PMC4474542; doi:10.1186/1471-2164-16-S7-S11)
Supplement: Additional file 5 — Figure S5. [file 1471-2164-16-S7-S11-S5.pdf]

Motif	p-value	Logo	TF
AAGCGCAA	<<1.0E-15		HNF4
GCGCGGCC	<<1.0E-15		E2F
CGCCGGGA	<<1.0E-15		AP2
AATCGGCT	<<1.0E-15		GATA1
CTCCGGTC	<<1.0E-15		AP4
CCGCGCTC	<<1.0E-15		E2F
CGCCGGAG	<<1.0E-15		AP2ALPHA
ATTCGGCC	<<1.0E-15		AIRE
CCGCGCCG	<<1.0E-15		E2F
ACCCGAAT	<<1.0E-15		PPARA
AAACGGCG	<<1.0E-15		MYB
AATCGGGT	<<1.0E-15		VDR
GCGCGAGC	<<1.0E-15		E2F1
CGGCGCAC	<<1.0E-15		HNF4
CGCCGAAG	<<1.0E-15		E2F
CCGCGGAA	<<1.0E-15		NRSE
CGTCGGAA	<<1.0E-15		ELK1
CGCCGTAA	<<1.0E-15		ELK1
GCGCGAAA	<<1.0E-15		E2F4DP1
AGCCGAAG	2.3E-13		PU1

(A) Active Promoter

Motif	p-value	Logo	TF
AGCCGCTG	<<1.0E-15		AP4
CAGCGGCC	<<1.0E-15		AP4
CCACGAGA	<<1.0E-15		HES1
CTGCGTCC	<<1.0E-15		WHN
CTCCGCAG	<<1.0E-15		NRSE
AGGCGGCC	<<1.0E-15		E2F
ATTCGGCC	<<1.0E-15		AIRE
CTCCGGCA	<<1.0E-15		AP2ALPHA
AGCCGAAG	<<1.0E-15		PU1
CAGCGGAG	<<1.0E-15		PAX5
AGCCGCCT	<<1.0E-15		E2F
AAGCGCAG	<<1.0E-15		HNF4
CTCCGAGC	<<1.0E-15		NRSE
ACCCGCAG	<<1.0E-15		AP2
CCTCGGAA	<<1.0E-15		NRSE
CCTCGTTC	<<1.0E-15		VDR
ACACGTAC	<<1.0E-15		HIF1
CACCGAGA	<<1.0E-15		CDPCR3
CTCCGGTC	<<1.0E-15		AP4
GGGCGGAA	<<1.0E-15		SP1

(B) Strong Enhancer

Figure S5 Sequence motifs surrounding mCpGs in regulatory regions. Additional 20 significant overrepresented motifs and their best matched TF binding sites are listed. (A) Active promoter (B) Strong enhancer.
